# Protective effect of phospholipids in lipoproteins against diabetic kidney disease: A Mendelian randomization analysis

**DOI:** 10.1371/journal.pone.0302485

**Published:** 2024-05-01

**Authors:** Tongyi Li, Liangliang Geng, Yunjiao Yang, Guannan Liu, Haichen Li, Cong Long, Qiu Chen

**Affiliations:** Hospital of Chengdu University of Traditional Chinese Medicine, Chengdu, Sichuan, China; Universita degli Studi di Milano, ITALY

## Abstract

**Background:**

The etiology of diabetic kidney disease is complex, and the role of lipoproteins and their lipid components in the development of the disease cannot be ignored. However, phospholipids are an essential component, and no Mendelian randomization studies have yet been conducted to examine potential causal associations between phospholipids and diabetic kidney disease.

**Methods:**

Relevant exposure and outcome datasets were obtained through the GWAS public database. The exposure datasets included various phospholipids, including those in LDL, IDL, VLDL, and HDL. IVW methods were the primary analytical approach. The accuracy of the results was validated by conducting heterogeneity, MR pleiotropy, and F-statistic tests. MR-PRESSO analysis was utilized to identify and exclude outliers.

**Results:**

Phospholipids in intermediate-density lipoprotein (OR: 0.8439; 95% CI: 0.7268–0.9798), phospholipids in large low- density lipoprotein (OR: 0.7913; 95% CI: 0.6703–0.9341), phospholipids in low- density lipoprotein (after removing outliers, OR: 0.788; 95% CI: 0.6698–0.9271), phospholipids in medium low- density lipoprotein (OR: 0.7682; 95% CI: 0.634–0.931), and phospholipids in small low-density lipoprotein (after removing outliers, OR: 0.8044; 95% CI: 0.6952–0.9309) were found to be protective factors.

**Conclusions:**

This study found that a higher proportion of phospholipids in intermediate-density lipoprotein and the various subfractions of low-density lipoprotein, including large LDL, medium LDL, and small LDL, is associated with a lower risk of developing diabetic kidney disease.

## Introduction

Diabetic kidney disease, a prevalent microvascular complication that manifests in patients with diabetes mellitus, has emerged as a predominant etiology of chronic kidney disease and end-stage renal disease in the diabetic population, owing to its pathogenesis as a diabetes-induced renal pathology [1, 2]. Research has shown that the overall quality and composition of various lipoproteins tend to remain relatively stable in individuals during the early stages of diabetic kidney disease [3]. It has been shown that the specific combination of phospholipids and free fatty acids can be used as a predictor of renal function decline in patients with diabetic kidney disease, and there have been numerous studies focusing on protective factors against and biomarkers of diabetic kidney disease [4, 5]. However, relatively few studies have focused on phospholipids. Therefore, the aim of our current study was to provide evidence of causal associations between phospholipids and a lower risk of diabetic kidney disease. Phospholipids, which are essential molecules composed of glycerol, fatty acids, and phosphate groups, are vital structural constituents of plasma membranes. However, recent research has unveiled their multifaceted roles as signaling molecules, exerting diverse physiological responses [6, 7]. Phospholipids are transported from the liver to other tissues by binding to specific proteins, and enzymes such as phosphodiesterases regulate the metabolism of phospholipids. Phospholipids, lipoproteins, and diabetic kidney disease exhibit a close interrelationship, necessitating further investigation into the underlying mechanisms and their clinical and research implications. Therefore, the hypothesis that the level of phospholipids in various lipoproteins can influence the development of diabetic kidney disease was proposed. To examine causality, MR analysis uses genetic variations as IVs, which provides marked advantages over other types of research designs [8]. Unfortunately, the causal association among phospholipids, lipoproteins, and diabetic kidney disease has not been investigated in Mendelian randomization studies. Consequently, this Mendelian randomization analysis was conducted to thoroughly examine the potential causal influence of phospholipids in lipoproteins on the pathogenesis and progression of diabetic kidney disease.

## Methods

Mendelian randomization analysis is predicated on the fulfillment of three fundamental assumptions. First, the single nucleotide polymorphisms (SNPs) utilized as IVs must exhibit a strong association with the exposure factors under investigation. Second, the SNPs employed as instrumental variables must only affect the outcome through the exposure factors. The selected IVs are only strongly associated with the exposure factors. Third, the instrumental variables must not be correlated with any confounding factors that could impact the relationship between the exposure and outcome factors. We used different statistical models and multiple methods to perform sensitivity and heterogeneity analyses to remove potential confounders. To enhance the robustness of the hypotheses and conclusions, the Steiger filtering method was utilized to eliminate confounding SNPs and examine the directional effect of the protective role. The genome-wide association study summary-level data employed in this study were accessed from the IEU Open GWAS project (https://gwas.mrcieu.ac.uk/). According to the policy of the Ethics Committee of the Hospital of Chengdu University of Traditional Chinese Medicine, this study was exempt from ethical review due to the utilization of publicly available data and complete anonymization of patient information.

### Data sources

The current study examined the role of phospholipids in lipoproteins as exposure factors for diabetic kidney disease. The data for diabetic kidney disease were extracted from the FinnGen [9] Biobank by the IEU Open GWAS project. The FinnGen GWAS diabetic kidney disease data fit the description of ICD-10 code N08.3* (glomerular disorders in diabetes mellitus). Additionally, the genome-wide association study summary-level data for the exposure factors of phospholipids in lipoproteins, specifically phospholipids in LDL, IDL, VLDL, and HDL, were accessed from the UK Biobank by the IEU Open GWAS project. Notably, proxy SNPs were not utilized in the identification of SNPs associated with the outcome.

### The selection of instrumental variables

In MR analysis, instrumental variables are used as intermediaries between exposure factors and outcomes to evaluate the causal association between the exposure factors and outcomes. Typically, genetic variations are used as IVs, with SNPs being the most commonly employed. To identify SNPs associated with phospholipids in lipoproteins, data were extracted from the IEU Open GWAS project. Subsequently, a rigorous screening process was conducted to identify single nucleotide polymorphisms demonstrating robust associations with the exposures of interest, with a genome-wide significance threshold of *P* < 5×10^−8^, a clumping window greater than 10,000 kb, and a linkage disequilibrium level of r^2^ < 0.001. This satisfied our association hypothesis. F-statistics and R^2^ were used to assess the statistical efficacy of the instrumental variables. F-statistics above 10 usually indicate the presence of a robust relationship between the IVs and the exposure factors as required by Mendelian randomization analyses [10].

### Statistical analysis

**Fig 1:**
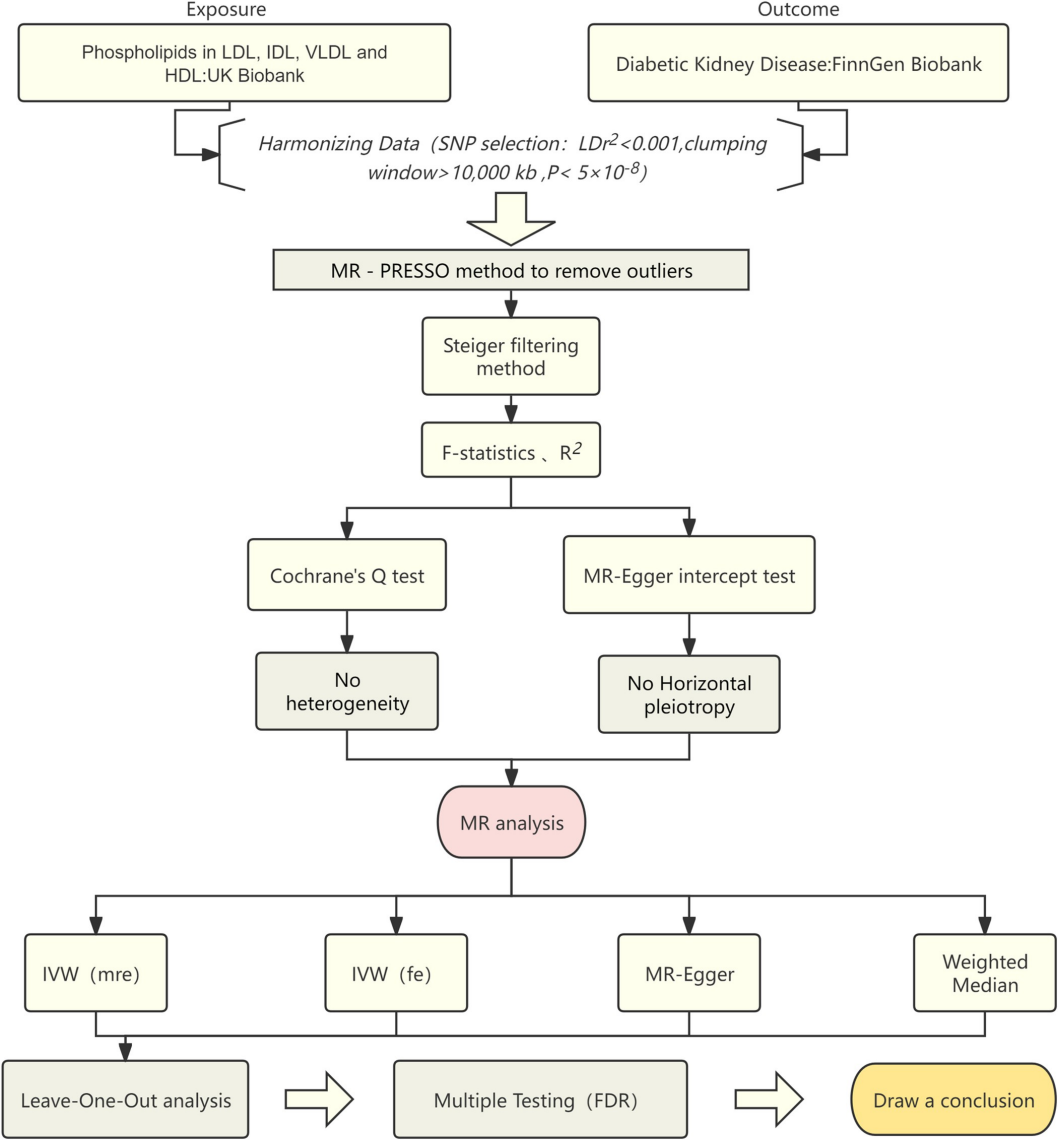
Flow chart of this Mendelian randomization study.

Causality was assessed through the inverse variance weighted approach, incorporating both fixed and multiplicative random effects. The IVW approach is regarded as the most reliable method for inferring causation in two-sample MR analyses if all assumptions of MR are met [11]. The processed data were analyzed using the IVW method and the weighted median and MR‒Egger methods. The MR‒Egger regression approach was employed to assess the potential presence of pleiotropic effects. Additionally, a leave-one-out analysis was conducted to examine the impact of outliers or pleiotropic genetic variants. Cochran’s Q test was utilized to estimate heterogeneity across all instrumental SNPs. To investigate the presence of potential pleiotropic effects, we employed the MR-PRESSO test to detect outlier single nucleotide polymorphisms (SNPs). In the MR-PRESSO test, the inverse variance weighting (IVW) method is applied through regression analysis, and the residual sum of squares is utilized as an indicator of heterogeneity. If the residual sum of squares exhibits a decrease relative to the expected distribution from simulations, the SNP in question is excluded from further analysis. To examine the causal direction between two attributes, the Steiger filtering method was employed. The MR analysis was then repeated to ensure the validity of the results. To ensure the accuracy of the analysis, samples still exhibiting pleiotropy were excluded from the study. Moreover, a leave-one-out analysis was conducted to evaluate whether the exclusion of any individual single nucleotide polymorphism markedly influenced the results. The P values obtained for statistically significant exposure factors were subjected to multiple testing correction by the false discovery rate (FDR) method. The IVW method (fixed and multiplicative random effects), the weighted median method, the MR‒Egger method and leave-one-out analyses were considered to indicate positive exposure factors if they were in the same direction. Fig 1 shows the flowchart for the analysis of this study. The “TwoSampleMR” and “MRPRESSO” packages in R Studio programming software were utilized to conduct all statistical analyses [12, 13].

## Results

**Fig 2:**
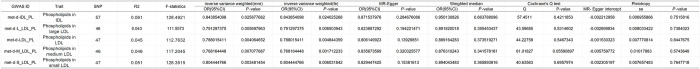
Positive results of Mendelian randomization analysis (after removing outliers by the MR-PRESSO test).

To investigate potential causal associations between phospholipids across diverse lipoproteins and diabetic kidney disease, 17 distinct exposure factors were examined. The number of SNPs utilized in this analysis ranged from 46 to 57, while the number of exposure factors was 115,078 individuals of European descent. These GWAS data were obtained from the UK Biobank developed by Nightingale Health, including males and females. The outcome factors comprised 3283 cases of diabetic kidney disease in male and female individuals of European ancestry derived from the FinnGen biobank, as well as 210,463 controls of European descent. For further details on the exposures and analysis results, please refer to Fig 2. Additional analyzed exposure nonpositive datasets can be found in S1 Table. Upon the removal of outliers, the F-statistic within this study ranged from 111.557 to 128.492, signifying that the instrumental variables employed in the analysis met the criteria for a robust association with the exposure factors under investigation. Additionally, we conducted a thorough reassessment of these exposure factors in relation to hypertensive renal disease (finn-b-I9_HYPTENSRD) and IgA nephropathy (ebi-a-GCST90018866) as outcome factors using MR analysis and determined that there was no causal association between them.

**Fig 3:**
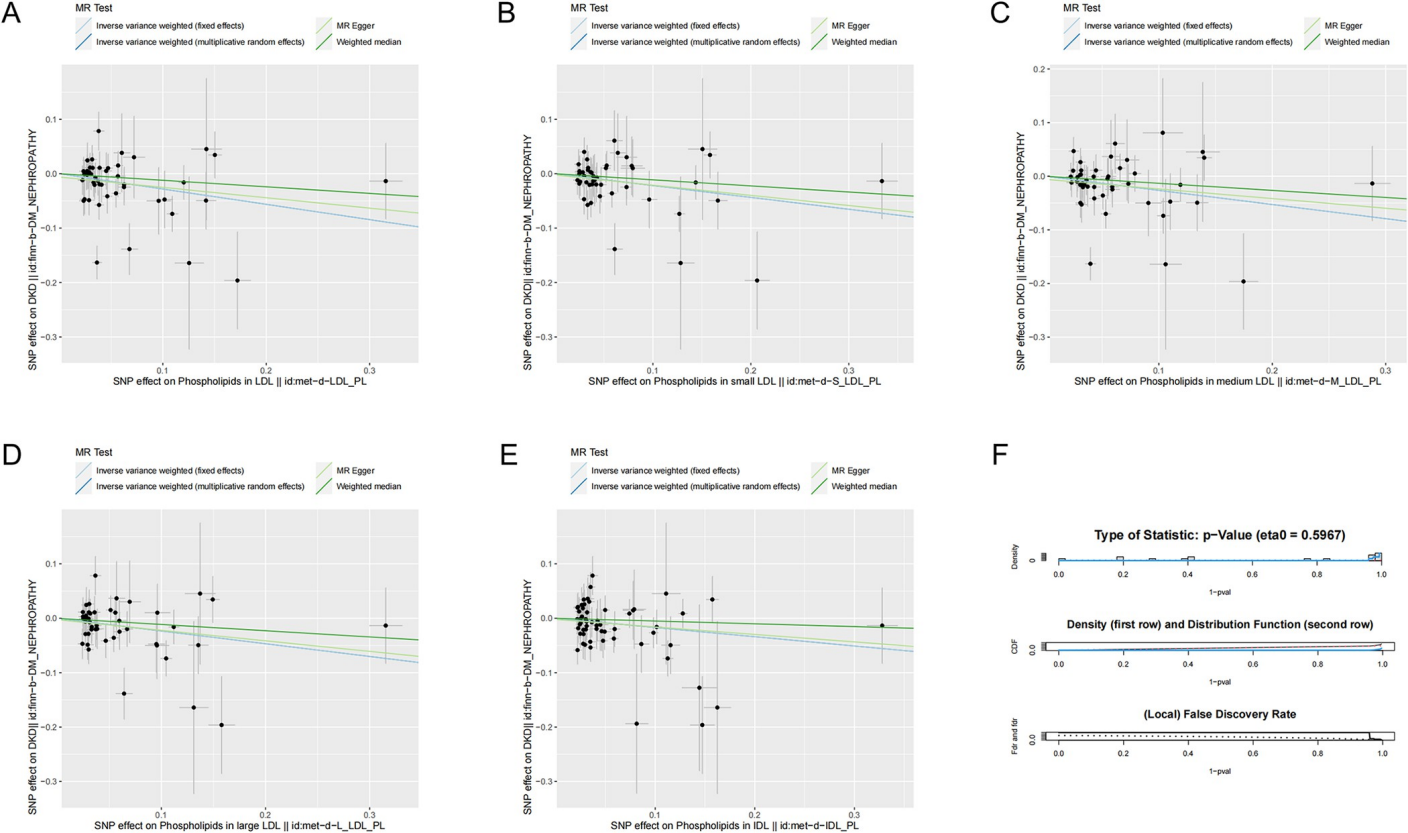
Scatterplot of the genetic association between phospholipids in lipoproteins and diabetic kidney disease. (A) Genetic association of phospholipids in LDL with diabetic kidney disease. (B) Genetic association of phospholipids in small LDL with diabetic kidney disease. (C) Genetic association of phospholipids in medium LDL with diabetic kidney disease. (D) Genetic association of phospholipids in large LDL with diabetic kidney disease. (E) Genetic association of phospholipids in IDL with diabetic kidney disease. (F) P value multiple testing correction by the FDR method.

**Fig 4:**
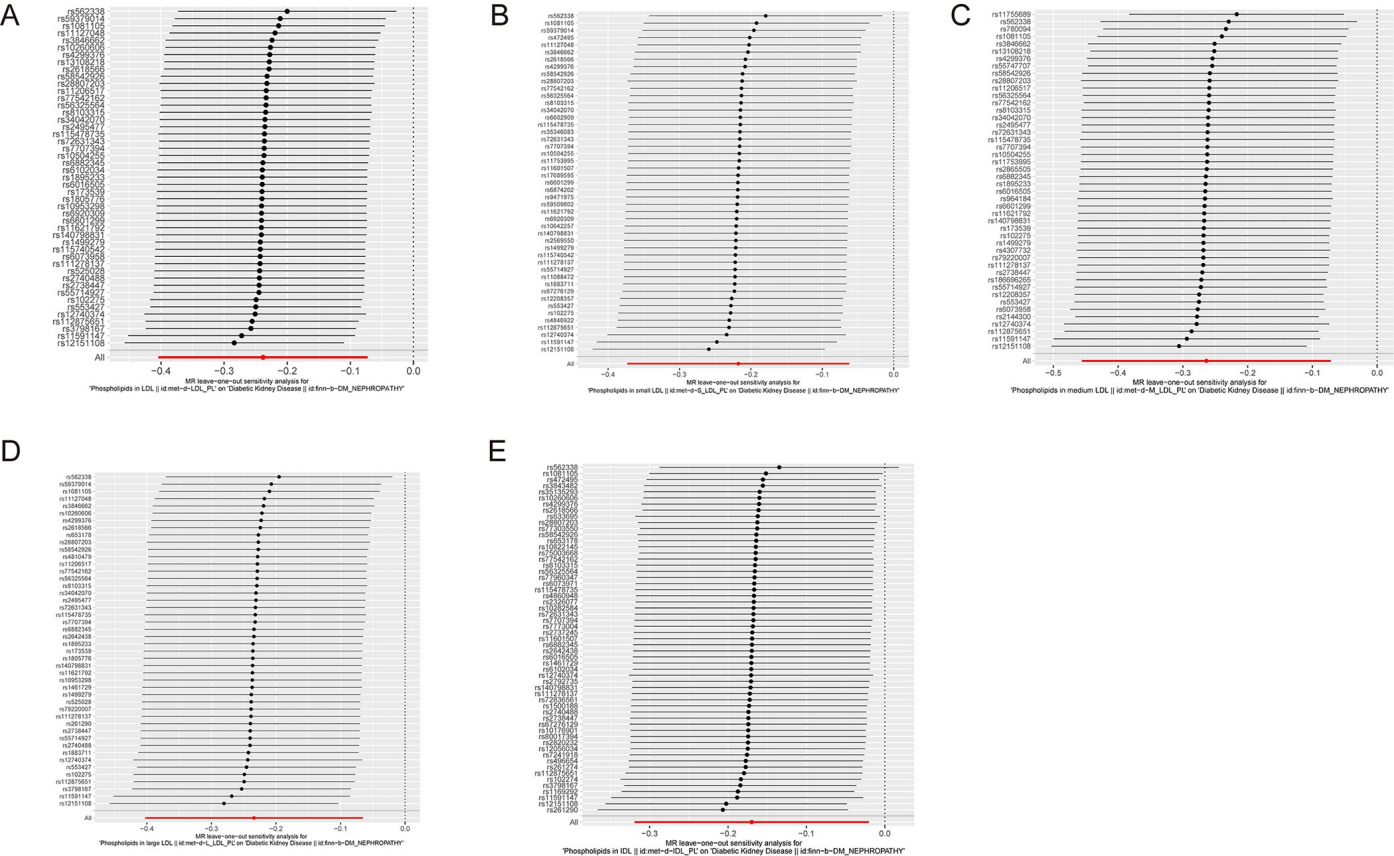
The results of leave-one-out analyses. (A) Genetic association of phospholipids in LDL with diabetic kidney disease (B) Genetic association of phospholipids in small LDL with diabetic kidney disease (C) Genetic association of phospholipids in medium LDL with diabetic kidney disease (D) Genetic association of phospholipids in large LDL with diabetic kidney disease (E) Genetic association of phospholipids in IDL with diabetic kidney disease.

In this study, a total of five protective exposure factors were identified after FDR multiple correction and the MR analysis described above (P < 0.05 by the IVW multiplicative random effects method). Phospholipids in intermediate-density lipoprotein (OR: 0.8439; 95% CI: 0.7268–0.9798; P = 0.025; FDR = 0.0412), phospholipids in large low-density lipoprotein (OR: 0.7913; 95% CI: 0.6703–0.9341; P = 0.006; FDR = 0.017), phospholipids in low-density lipoprotein (after removing outliers, OR: 0.788; 95% CI: 0.6698–0.9271; P = 0.004; FDR = 0.017), phospholipids in medium low-density lipoprotein (OR: 0.7682; 95% CI: 0.634–0.931; P = 0.007; FDR = 0.017), and phospholipids in small low-density lipoprotein (after removing outliers, OR: 0.8044; 95% CI: 0.6952–0.9309; P = 0.0034; FDR = 0.017) were discovered as protective factors. However, the results of MR‒Egger analyses were not statistically significant (P>0.05). The scatter plot of the MR analysis results is shown in Fig 3. The presence of potential horizontal pleiotropy was not observed (NbDistribution = 1000, global test > 0.05), and Cochran’s Q test did not find heterogeneity after removing outliers by the MR-PRESSO test for IVs in exposure factors (P > 0.05). Horizontal pleiotropy was still detected in the MR‒Egger intercept test upon analysis of the dataset with GWAS ID met-d-XL_HDL_PL (phospholipids in very large HDL) after MR-PRESSO analysis (P <0.05). As a result, this dataset was excluded from the analysis results. Notably, leave-one-out analysis demonstrated that the detected causal associations remained robust, suggesting that the identified protective factors were reliable and consistent (Fig 4). The causal relationship identified through the MR analysis is indicative of the impacts arising from prolonged exposure to associated factors. In terms of univariate MR analyses, the observed effects primarily pertain to the overall associations between exposures and outcomes, rather than direct effects specifically linking exposures and outcomes. It is worth noting that the interplay between exposures and outcomes can involve intricate and multifaceted mechanisms. The results of this study suggest that a higher proportion of phospholipids in intermediate-density lipoprotein and the various subfractions of low-density lipoprotein, including large LDL, medium LDL, and small LDL, is associated with a lower risk of developing diabetic kidney disease.

## Discussion

Early stages of DKD are characterized by dyslipidemia, platelet dysfunction, and albuminuria, all of which independently potentiate the pathogenesis and advancement of vascular injury [14, 15]. Early-stage diabetic kidney disease can exert an influence on lipoprotein metabolism through its impact on urinary lipid excretion or by inducing secondary effects on hepatic production and lipoprotein clearance. However, it is noteworthy that the majority of studies investigating the temporal pattern of diabetic kidney disease have primarily focused on glycemia, clinical symptoms, or specific biomarkers, thereby limiting the understanding of the intricate metabolic phenotype associated with lipid metabolism in this context [16]. This intricate interplay between phospholipids and their associated signaling pathways contributes to their significant impact on cellular function and overall physiological homeostasis. For instance, lysophosphatidic acid, a naturally occurring small molecule produced from glycerophospholipids, is synthesized through the enzyme activity of lysophospholipases such as autotaxin and phospholipases A1 and A2. Recent studies suggest that ligand binding to lysophosphatidic acid (LPA) receptors may contribute significantly to the pathogenesis of diabetic kidney disease [6]. By Mendelian randomization analysis, the evidence suggests that phospholipids in IDL and various LDL subfractions are associated with a protective effect against diabetic kidney disease. Numerous findings have also pointed to a critical role of phospholipids in the pathological mechanisms underlying diabetic kidney disease, particularly those within the different LDL subfractions. Additionally, it is worth noting that the phospholipid composition of these lipoprotein particles may be influenced by the increased activity of lipid transfer proteins observed in diabetic patients [17]. Lipid metabolism is also impacted in patients with other forms of chronic kidney disease. However, the specific lipid abnormalities may vary depending on the underlying etiology of the renal condition. Hence, we conducted an MR analysis involving 17 exposure factors as independent variables and IgA nephropathy and hypertensive nephropathy as outcome factors, yet no causal relationship was established. This underscores the specificity of our conclusion regarding diabetic kidney disease and its limited applicability to other types of chronic kidney diseases. Enzymatic fluorometric assays have yielded preliminary findings indicating distinct variations in the phospholipid composition of VLDL, LDL, and HDL [18]. These observations underscore the significance of quantifying the phospholipid component within each lipoprotein fraction. The findings receive partial support from clinical studies conducted on four independent cohorts comprising both type 2 diabetic and nondiabetic individuals. These studies revealed a positive association between phospholipids in LDL and cholesterol esters and the estimated eGFR in patients diagnosed with type 2 diabetes, suggesting a potentially favorable impact on kidney function [17]. In the case of acute kidney injury, LPA can produce an overall effect of kidney cell protection [7, 19]. Okusa et al.’s study also showed that LPA2 protects the kidney against ischemic injury [20]. However, it is crucial to acknowledge the intricate and multifaceted nature of the pathogenesis underlying diabetic kidney disease. Therefore, it becomes imperative to identify precise cause-and-effect relationships that can provide valuable insights to guide further research endeavors and inform clinical practice. Evidence suggests that the use of antilipemic agents to reduce lipid levels may have a beneficial effect on the glomerular filtration rate and proteinuria in patients with diabetes. For instance, simvastatin was found to decrease the rate of major vascular events and reduce the glomerular filtration rate by 25% in these individuals [21, 22]. Therefore, investigating the mechanisms underlying lipid accumulation and potential lipotoxicity in the human kidney could represent a promising avenue for delaying the onset and progression of diabetic kidney disease [23]. Diabetic and nondiabetic individuals exhibit distinct lipid metabolic processes, leading to variations in lipoprotein composition and prognostic implications [17]. The augmentation of LDL mass in the early stages and subsequent diagnosis of diabetic kidney disease primarily stems from the increased number of LDL particles. However, the elevated LDL mass observed in patients with proteinuric insulin-dependent diabetes mellitus is predominantly attributed to a rise in light LDL [24]. Individuals with dyslipidemia, a consequence of chronic kidney disease, have unique characteristics that deviate from those observed in the general populace. Beyond quantitative alterations, patients with renal disease exhibit qualitative lipid modifications that remain undetectable via conventional testing methodologies [25, 26]. Consequently, there is an imperative need to delve deeper into the clinical application of lipid modifications in diabetic kidney disease patients.

Diabetic kidney disease, while often categorized as a vascular condition, has an underlying pathogenesis that remains elusive. Diabetic kidney disease can be divided into normoalbuminuric diabetic kidney disease (NADKD) and proteinuric diabetic kidney disease. The DKD diagnostic criteria proposed by the American Diabetes Association (ADA) in 2015 include a UAER >30 mg/24 h or an eGFR < 60 ml/(min·1.73 m^2^) [27]. Similarly, dyslipidemia is considered a risk factor for NADKD [28]. The current GWAS on diabetic kidney disease did not categorize this. There are fewer clinical studies in this area, but the protective factors identified in our results should also apply to NADKD. The majority of contemporary studies investigating the role of lipid metabolism in DKD have primarily concentrated on the immune-inflammatory response and oxidative stress mechanisms. For example, within the arterial wall, sphingomyelinase catalyzes the hydrolysis of sphingomyelin to ceramide within LDL, thereby enhancing macrophage uptake of LDL. This process culminates in the formation of foam cells, a hallmark of atherosclerosis [29]. While LDL is traditionally recognized as the principal source of extracellular cholesterol, it also serves as a significant reservoir of extracellular phospholipids. Platelet-activating factor mimetics and other phospholipid oxidation products are present in atherosclerotic lesions [30]. LDL-derived oxidized phospholipids play a pivotal role as constituents of the nonspecific innate immune system [31]. Extensive scientific evidence has firmly established the profound impact of lipoprotein and phospholipid oxidation on the induction of a diverse spectrum of immune responses in vivo. Notably, the process of lipid peroxidation gives rise to the formation of reactive aldehydes and oxidized phospholipids, which intricately engage in the formation of complex immune adducts with proteins or other phospholipids [32]. In contrast, interventions can affect the immune response to oxidized LDL and reduce the progression of atherosclerosis in animal models [32]. The provision of supplementary seed molecules by vascular wall cells is crucial for circulating LDL to attain the requisite threshold concentration for phospholipid oxidation [33]. The equilibrium between noninflammatory LDL and its mildly oxidized counterpart may serve as a determinant, albeit not a dominant one, in the vulnerability of the vascular system. This interplay could influence the progression or deceleration of diabetic kidney disease, a complex renal complication of diabetes. However, the precise role and significance of associated lipids and lipoproteins in this context warrant further rigorous scientific exploration.

The strength of this study is that it assessed whether a comparatively high proportion of phospholipids in different lipoproteins is a protective factor against the development of diabetic kidney disease in the European population. Our research uncovered potential clinical implications. First, regular lipid profiling in diabetic patients, including a detailed analysis of IDL and LDL subfractions, could become an essential component of routine health assessments. Second, nutritional guidance aimed at modulating the lipid composition of diets may be provided to patients and health care providers. For example, omega-3 phospholipids have proven efficacy in improving impaired glucose homeostasis and insulin sensitivity in obese patients, and krill oil can be a major source of omega-3 phospholipid intake [34]. Last, understanding individual lipid profiles could pave the way for personalized medicine approaches. The regular monitoring of lipid profiles in diabetic patients, particularly those displaying early signs of kidney dysfunction, could facilitate the timely and effective adjustment of therapeutic strategies. This insight may open avenues for further research into the development of new therapeutic agents targeting lipid metabolism specifically related to diabetic kidney disease. Although the results vary among populations, this study provides new directions for precise treatment in European populations. Bias due to potential confounders can be greatly reduced through MR analysis, and the impact on study conclusions caused by reverse causality can also be avoided. By studying a homogeneous population, we aimed to reduce potential biases and increase the internal validity of our study. It is also important to consider that genetic studies in diverse populations can present unique challenges due to population stratification and differences in allele frequencies. However, this study was relatively preliminary, and the positive effects of phospholipids in different lipoproteins on the population with diabetic kidney disease need to be further investigated. Further research on ways to elevate the levels of phospholipids in relevant lipoproteins in people at risk for the disease is also needed. In addition, this study established some causal relationships from a genetically based perspective, although it is necessary to conduct clinical cohort studies to further validate the findings.

## Conclusion

This study found that a higher proportion of phospholipids in intermediate-density lipoprotein and the various subfractions of low-density lipoprotein, including large LDL, medium LDL, and small LDL, is associated with a lower risk of developing diabetic kidney disease. This finding can assist clinical physicians in risk management and prognosis evaluation for certain high-risk patients by identifying phospholipid levels in lipoproteins, particularly in diabetic patients. Increased levels of phospholipids in these types of lipoproteins after a period of intervention may have a positive effect on reducing the incidence of diabetic kidney disease. Furthermore, further quantitative and mechanistic studies on the various lipid components of various lipoproteins will have a positive effect on the clinical management of diabetic kidney disease.

## Supporting information

S1 Table Other analyzed exposure nonpositive datasets. (XLSX)

## Abbreviations

DKD: Diabetic kidney disease
VLDL: Very low-density lipoprotein
IDL: Intermediate-density lipoprotein
LDL: Low-Density Lipoprotein
MR: Mendelian randomization
IVs: Instrumental variables
SNPs: Single nucleotide polymorphisms
HDL: High-Density Lipoprotein
IVW: Inverse-variance weighted
GWAS: Genome-wide association studies
OR: Odds ratio
CI: Confidence interval
eGFR: estimated Glomerular filtration rate
T2DM: Type 2 diabetes mellitus
